# Use of Fluorescent 2-AB to Explore the Bidirectional Transport Mechanism of *Pseudostellaria heterophylla* Polysaccharides across Caco-2 Cells

**DOI:** 10.3390/molecules27103192

**Published:** 2022-05-17

**Authors:** Bin Yang, Yuan Li, Wentao Shi, Yingying Liu, Yongjun Kan, Jinlong Chen, Juan Hu, Wensheng Pang

**Affiliations:** 1 The Second Affiliated Hospital of Fujian University of Traditional Chinese Medicine, Fuzhou 350003, China; 13905915817@163.com (B.Y.); ly13598052688@126.com (Y.L.); 13860662716@163.com (W.S.); liuyy0591@163.com (Y.L.); 2 School of Pharmacy, Fujian University of Traditional Chinese Medicine, Fuzhou 350122, China; jinlongchen9@126.com; 3 Institute of Materia, Fujian Academy of Traditional Chinese Medicine, Fuzhou 350003, China; kanyongjun@126.com

**Keywords:** *Pseudostellaria heterophylla* polysaccharides, 2-AB fluorescent labeling, HPLC-FLD, bi-directional transport, caco-2 cell

## Abstract

Polysaccharides are abundant in natural resources and perform numerous physiological functions. Polysaccharide structures often lack chromophore groups; thus, current analytical methods cannot distinguish polysaccharide metabolites in the body or polysaccharide prototypes in biological samples. Thus, the measurement of polysaccharides in blood, bodily fluid, and cell-culture medium is difficult. Our early-stage research resulted in the isolation of two homogeneous polysaccharides from *Pseudostellaria heterophylla*, PHP_0.5MSC-F_ and PHP_H-1-2_, which have anti-hyperglycemia and insulin resistance improvement effects for type 2 diabetes. In this study, the reducing terminal sugars of PHP_0.5MSC-F_ and PHP_H-1-2_ were labeled with 2-aminobenzamide (2-AB) to prepare novel fluorescent probes for HPLC-coupled fluorescence detection (HPLC-FLD). Quantitative analysis was performed in reference to T40, and the detection limit for PHP_0.5MSC-F_ was found to be 8.84 μg/mL with a linear range of 29.45–683.28 μg/mL. In reference to T70, the detection limit for PHP_H-1-2_ was found to be 13.89 μg/mL with a linear range of 46.29–462.76 μg/mL. This method was used to measure the bidirectional transport of polysaccharides across caco-2 cells from apical to basolateral (AP→BL) or from basolateral to apical (BL→AP) directions and to evaluate the polysaccharide bioavailability. The drug absorption capacity was determined based on the apparent permeability coefficient (Papp), and the Papp values for the two polysaccharides were found to be greater than 1 × 10^−6^ cm/s, which suggests easy absorption. Regarding bidirectional transport, the AP→BL Papp values were greater than the BL→AP values; thus, PHP_0.5MSC-F_ and PHP_H-1-2_ mainly underwent passive transference. The two membrane permeable polysaccharides were not P-gp efflux transporter substrates. The absorption mechanism of PHP_0.5MSC-F_ complies with passive diffusion under a concentration gradient, whereas PHP_H-1-2_ mainly utilizes a clathrin-mediated endocytic pathway to enter caco-2 cells. This innovative HPLC-FLD method can help to track polysaccharide internalization in vitro and in vivo to facilitate cellular uptake and biodistribution exploration.

## 1. Introduction

Polysaccharides are abundant constituents of living organisms, including animals and plants, and are widely used in Chinese traditional medicine [1]. Polysaccharides perform several physiological functions, including immune enhancement and antivirus, antitumor, and antiaging activities [2,3,4]. *Pseudostellaria heterophylla* (PHP) (Miq.) Pax ex Pax et Hoffm. is included as a medicine in the Pharmacopoeia of the People’s Republic of China [5]. *Pseudostellaria heterophylla* contains a high content of polysaccharides that can moisten the lungs, decrease coughing, and decrease blood sugar, blood lipids, and cholesterol. Notably, PHP polysaccharides can improve insulin resistance in type 2 diabetes [6,7,8,9].

Our previous studies identified two water-soluble homogeneous polysaccharides from PHP. One pectic polysaccharide (0.5MSC-F), with a molecular weight of 4.8 × 10^4^ Da, was found to be composed of rhamnose, galactose, arabinose, and galacturonic acid and was shown to stimulate insulin secretion [10]. The other was a glucan, comprised of only D-glucose (H-1-2), with a mean molecular weight of 1.4 × 10^5^ Da, which was found to increase glucose uptake and utilization in muscle and adipose cells [11].

The quantification of polysaccharides in vivo is crucial for studying their pharmacokinetics and conducting clinical monitoring. However, polysaccharide structures often lack chromophore groups that can be detected using high performance liquid chromatography (HPLC) by measuring scattering and refractive indexes or through phenol-sulfuric acid spectrophotometry [12]. The sensitivities of these methods are relatively poor, and polysaccharide content tests are less accurate when the analytical target is disturbed by a complex matrix. A precolumn derivatization reagent was developed for the analysis of polysaccharide monosaccharide compositions using an HPLC method with an ultraviolet or fluorescence detector. These methods have high sensitivity but fail to clearly differentiate polysaccharide metabolites from the prototypes [13]. Besides, even when the above methods are used, the absorption of polysaccharides orally as complete molecules cannot be directly detected in vivo, and thus, the polysaccharide detection method requires further investigation.

Fluorescence techniques have many advantages, such as high sensitivity, high selectivity, and convenience [14]. These techniques have been used for the detection of *Lycium barbarum* polysaccharides in rat plasma [15], rice bran, and ginseng polysaccharide uptake in cell lines [16,17], *Ganoderma applanatum* polysaccharide cellular localization in human colorectal cancer SWWC1116 cells [18], and the oral absorption characteristics of a pectin-type polysaccharide from *Smilax china* [19].

All sugar chains with the reducing end of the hemiacetal hydroxyl group can be UV-Vis or fluorescence derivatized using the reductive amination reaction (Schiff’s base reaction). Commonly used UV-Vis and fluorescence derived reagents include p-aminobenzoic acid ethyl ester (ABEE), acetaminacridone (AA-Ac), 8-aminonaphthalene-1,3,6-trisulfonic acid (ANTS), 2-aminopyridine (2-AP), and 2-aminobenzamide (2-AB). Among these, 2-AP and 2-AB are widely used and are available in a commercialized labeling kit with an established standard sugar spectrum [20,21]. Multimode chromatographic methods, capillary electrophoresis, and high-pH anion-exchange chromatography with pulsed amperometric detection are established methods for isolating and purifying labeled oligosaccharides [22].

Caco-2 cell monolayers derived from human colon adenocarcinoma have been widely used in drug research and development for estimating intestinal absorption and transportation characteristics. Notably, there is a good correlation between drug transport through caco-2 cell monolayers and in vivo absorption [23].

In this study, the reducing terminal sugars of two polysaccharides from PHP were labeled with 2-AB to develop a novel fluorescent probe for HPLC-coupled fluorescence detection (HPLC-FLD). This method was used to measure the bidirectional transport of polysaccharides across caco-2 cells in the apical to basolateral (AP→BL) and basolateral to apical (BL→AP) directions and to evaluate the polysaccharide bioavailability.

## 2. Results

### 2.1. Polysaccharide-2-AB

**Figure 1:**
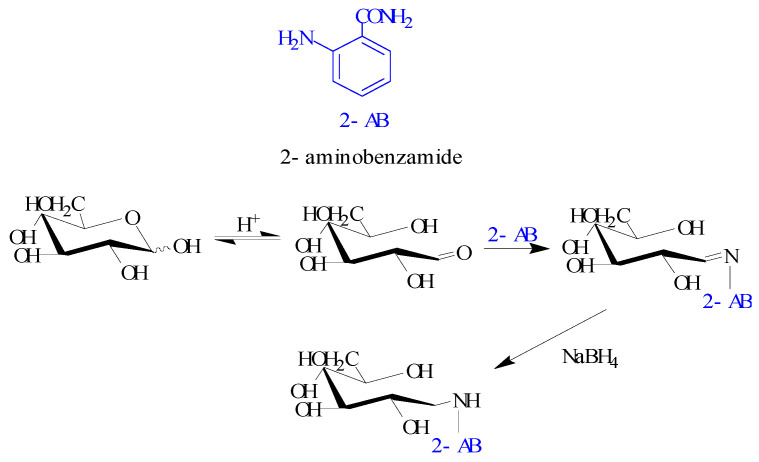
Schiff base reaction of 2-AB with glycan.

Semialdehyde groups (R-O-R’-OH) were formed by hydroxyl 5 and 1, the latter of which was at the reducing terminus of the monosaccharide. The open and closed loops existed in temporarily stable equilibrium states, and thus, a certain proportion of the reducing aldehyde group was present in the solution. The Schiff base reaction occurred between the aldehyde groups on the sugar and amino groups on 2-AB under certain conditions. The Schiff’s base reaction is a group-specific reaction for aldehydes, and 2-AB labels glycans that contain a free reducing terminus with high efficiency [17,18,19]. Reducing sugars can be visualized with fluorescent 2-AB through the Schiff base reaction, and a simplified reaction scheme for this type of reaction is shown in Figure 1.

**Figure 2:**
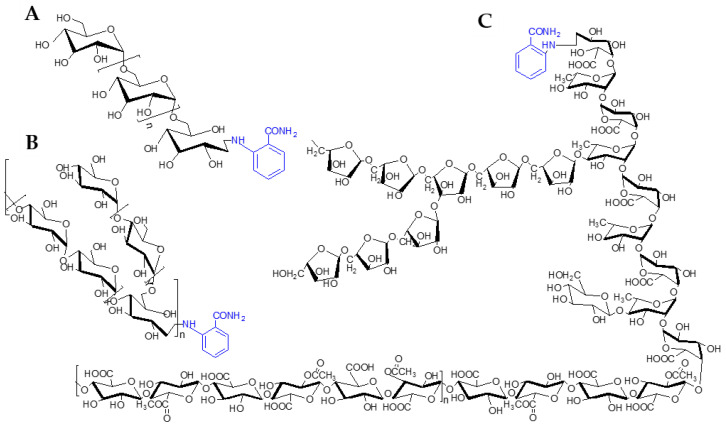
Structures of derivatized fluorescent polysaccharides. Blue highlights the structure of 2-AB in the figure. Dextran derivatives with 2-AB (**A**), PHP_H-1-2_-2AB (**B**), and PHP_0.5MSC-F_-2-AB (**C**) are shown.

Two fluorescent polysaccharides, PHP_H-1-2_-2AB and PHP_0.5MSC-F_-2-AB, were synthesized upon nucleophilic addition at the reducing terminus of PHP_H-1-2_ glucose or PHP_0.5MSC-F_ glucuronic acid with 2-AB and the reduction of the double bonds in the Schiff base NaBH_4_. The structures of the dextran reference substance, polysaccharide 0.5MSC-F, and H-1-2 derivatized with 2-AB are shown in Figure 2.

The substitution degrees of 2-AB for PHP_H-1-2_ and PHP_0.5MSC-F_ were 7.31 mg/g and 0.55 mg/g, respectively. Meanwhile, the stability studies of the two labeled polysaccharides in vitro showed that PHP_H-1-2_-2AB and PHP_0.5MSC-F_-2-AB were stable in PBS solution, simulated gastric fluid, and simulated intestinal fluid.

### 2.2. Polysaccharide-2-AB Analysis by Thin Layer Chromatography

**Figure 3:**
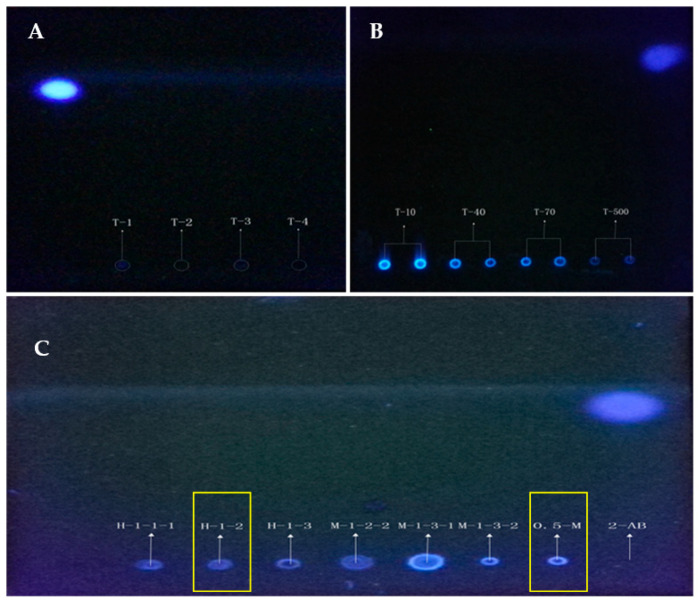
TLC identification method for polysaccharide-2-AB. T-1 and T-2 are PHP_H-1-2_ and PHP_0.5MSC-F_, T-3 and T-4 are NaBH_4_ and the blank solvent (**A**). Fluorescence spots with dextran T-500, T-70, T-40 and T-10 derivatives with 2-AB are shown (**B**). Fluorescence spots of 7 kinds of polysaccharide-2-AB are shown, and the yellow rectangle display shows the study of PHP_H-1-2_-2AB and PHP_0.5MSC-F_-2-AB presented in this article (**C**). In every one of the TCL plates, the uppermost blue spots are all 2-AB fluorescence.

The two types of polysaccharide-2-AB were shown to be highly polar and were easily soluble in water. Several highly polar solvents were tested as the moving phase, showing a serious peak tailing phenomenon. Using UV light at a wavelength of 320 nm, 2-AB was found to be soluble in the propanol-aqueous (3:1) phase, while the polysaccharide-2-AB was found to be insoluble. This allowed the free 2-AB to move up the thin-layer chromatography (TLC) plate while the labeled polysaccharide remained fixed at the initial position. The fluorescent spots revealed the presence of fluorescently labeled polysaccharide. The TLC plates were photographed (Figure 3).

### 2.3. Polysaccharide-2-AB Purification and Molecular Weight Determination

The products of the fluorescence labeling reaction were washed with 40 mL of methanol and then centrifuged for 10 min at 3000 rpm to remove the supernatant. The precipitate was dried to remove methanol at 40 °C. We repeated this process three times, until we could not detect 2-AB in the supernatant. Most impurities were removed by a HiTrap desalting column with 5 mL capacity prior to elution with water.

The molecular weight was calculated using the following equation [24]:

log*M* = a + b*Rt*

Here, *M* is the known molecular weight of the sample. *Rt* is the retention time of the sample. Chromatographic peaks assigned to polysaccharides were identified by the retention time and by comparison with the standards.

**Figure 4:**
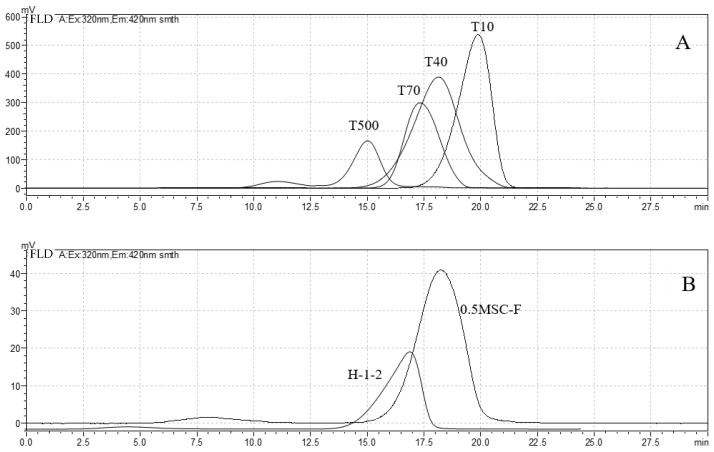
Gel exclusion chromatogram of 4 dextran standards and 2 polysaccharide samples with 2-AB fluorescence labeling. Dextran derivatives with 2-AB (**A**) and PHP_H-1-2_-2AB and PHP_0.5MSC-F_-2-AB (**B**) are shown.

By injecting 20 μL of 240 μg/mL, four fluorescently labeled dextran standards (dextran T10, T40, T70, and T500) were determined by gel exclusion chromatography (GPC). A plot of the results is shown in Figure 4, and the results of *Rt* are shown in Table 1. Standard curves were prepared using Empower GPC software. The linear regression equation was log*M* = -0.356 *Rt* + 11.08. The correlation coefficient was 0.9965, and log*M* was inversely related to *Rt*. PHP_0.5MSC-F_-2-AB was retained in the column for 18.03 min, and its molecular weight was estimated to be about 46,355 Da. PHP_H-1-2_-2AB was retained in the column for 16.49 min, and its molecular weight was estimated to be about 161,770 Da. The larger the molecular weight of a polysaccharide was, the shorter the retention time was.

### 2.4. Polysaccharide-2-AB Analysis by HPLC-FLD

Two polysaccharide-2-AB solutions and two reference substances, dextran T40-2-AB and dextran T70-2-AB, were prepared in concentrations of 240 μg/mL. The two dextrans were selected as standards due to their similar molecular weights and high detection sensitivity levels.

**Figure 5:**
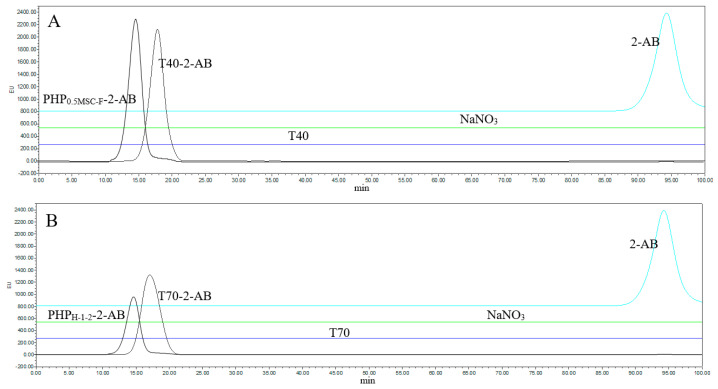
The HPLC chromatogram of dextran standards and polysaccharide samples with 2-AB fluorescence labeling: (**A**) PHP_0.5MSC-F_-2-AB (240 µg/mL); (**B**) PHP_H-1-2_-2-AB (240 µg/mL).

The HPLC-FLD method was established for the quantitative evaluation of polysaccharide-2-AB. In the mobile phase (NaNO_3_) solution, T40 and T70 could not emit fluorescence when exposed to UV light. However, PHP_0.5MSC-F_-2-AB, PHP_H-1-2_-2-AB, and 2-AB presented characteristic fluorescence peaks in the chromatogram with maximum concentrations at 13.5 min, 14.4 min, and 94.0 min, respectively. A quantitative analysis of PHP_0.5MSC-F_-2-AB was performed in reference to T40-2-AB, and the detection limit for PHP_0.5MSC-F_-2-AB was found to be 8.84 μg/mL with a linear range of 29.45–683.28 μg/mL (Figure 5A). A quantitative analysis of PHP_H-1-2_-2-AB was performed in reference to T70-2-AB, and the detection limit for PHP_H-1-2_-2-AB was found to be 13.89 μg/mL with a linear range of 46.29–462.76 μg/mL (Figure 5B). The above results confirm that the method is of sufficient sensitivity for the evaluation of PHP_0.5MSC-F_-2-AB and PHP_H-1-2_-2-AB.

### 2.5. The Bidirectional Transport of Polysaccharides

The effect of polysaccharide-2-AB on cellular viability was determined using the 3-(4.5-dimethyl-2-thiazoly1)-2, 5-diphenyl-2H-terazolium bromide (MTT) method [10,11]. Caco-2 cell monolayers were incubated with PHP_0.5MSC-F_-2-AB or PHP_H-1-2_-2-AB at different doses for 12, 24, and 48 h. The cellular activity was negatively correlated with the concentration of PHP_0.5MSC-F_-2-AB versus that of PHP_H-1-2_-2-AB. Compared to that in the control group, cellular activity decreased by over 10% (*p* < 0.01) when the concentration of PHP_0.5MSC-F_-2-AB and PHP_H-1-2_-2-AB was greater than 550 μg/mL.

The caco-2 cell monolayers were incubated with different concentrations of PHP_0.5MSC-F_-2-AB or PHP_H-1-2_-2-AB (100 μg/mL, 200 μg/mL, 400 μg/mL) for different durations within two hours. To investigate polysaccharide transport from AP to BL, 100 μL of liquid was sampled from BL and replenished with 100 μL of HBSS solution. BL to AP sampling was performed similarly. Samples were centrifuged at 1000 rpm for 5 min at 4 °C and stored in a −80 °C refrigerator.

The cell fluid polysaccharide concentrations were determined at different time points (30 min, 60 min, and 120 min) using the HPLC-FLD method. The apparent permeability coefficient (Papp) of the two polysaccharides was calculated as follows [18]:

Papp (cm/s) = ΔQ/(Δt·A·C_0_)

where ΔQ (μg) is the transport amount over time (Δt (s)), A (cm^2^) is the membrane area, and C_0_ (μg/mL) is the initial polysaccharide concentration on the AP side of the caco-2 cell monolayer.

The Papp values for the two polysaccharides were greater than 1 × 10^−6^ cm/s, which suggests that both were able to permeate the caco-2 monolayer. As the concentration of polysaccharides and incubation time increased, the accumulative transport also increased. However, as the time increased from 30 min to 120 min, Papp began to decline. This might be due to the increased exposure time, which resulted in the saturation of the monolayer surface with polysaccharides and a decrease in the monolayer permeability.

To investigate the bidirectional transport of polysaccharides in the AP→BL and BL→AP directions, the growth medium was removed from the basolateral or apical compartments of the Transwell plate. PHP_0.5MSC-F_ and PHP_H-1-2_ presented higher AP→BL Papp than BL→AP Papp values, suggesting that the transport modes of PHP_0.5MSC-F_ and PHP_H-1-2_ were through passive transference. At concentrations of 100 μg/mL, 200 μg/mL, and 400 μg/mL, the PHP_H-1-2_ Papp values were greater than those of PHP_0.5MSC-F_ in multiple time intervals. Notably, at 100 μg/mL, the PHP_H-1-2_ Papp values were 1.5 times greater than those of PHP_0.5MSC-F_ at 30 min. The Papp difference between the two polysaccharides decreased as concentration and time increased. The permeability and absorption ability of PHP_H-1-2_ were greater than those of PHP_0.5MSC-F_. Detailed accumulative transport and apparent permeability coefficient information for PHP_0.5MSC-F_ and PHP_H-1-2_ is displayed in Table 2.

### 2.6. The Influences of Verapamil, Chlorpromazine, Nystatin, Amiloride, and P-gp Efflux Pump Inhibition on Polysaccharide Transport

The transport of PHP_0.5MSC-F_ and PHP_H-1-2_ in the AP→BL direction was measured in the absence and presence of verapamil (P-gp inhibitor). The Papp (×10^−5^ cm/s) values for 200 μg/mL PHP_0.5MSC-F_ in the AP→BL direction were 31.32 ± 2.62, 21.65 ± 3.16, and 14.01 ± 3.69 at 30 min, 60 min, and 120 min, respectively, and 35.52 ± 3.14, 19.35 ± 2.68, and 12.41 ± 3.27 in the presence of verapamil. These differences were not found to be statistically significant (*p* > 0.05) (Figure 5A). The Papp (×10^−5^ cm/s) values for 200 μg/mL PHP_H-1-2_ in the AP→BL direction were 44.80 ± 4.41, 26.71 ± 4.05, and 14.78 ± 4.73 at 30 min, 60 min, and 120 min, respectively, and 44.62 ± 4.39, 25.79 ± 3.91, and 13.31 ± 4.26 in the presence of verapamil. The differences were not found to be statistically significant (*p* > 0.05) (Figure 5B).

### 2.7. Endocytosis Pathways of PHP_0.5MSC-F_ and PHP_H-1-2_

**Figure 6:**
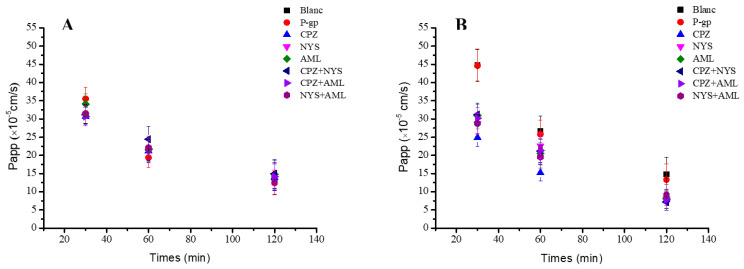
Verapamil, Chlorpromazine, Nystatin, Amiloride, and two of the three affected during transport of PHP_0.5MSC-F_ and PHP_H-1-2_ in the AP→BL direction at different times of 30 min, 60 min, and 120 min: (**A**) PHP_0.5MSC-F_ (200 µg/mL); (**B**) PHP_H-1-2_ (200 µg/mL).

The AP→BL transport of PHP_0.5MSC-F_ and PHP_H-1-2_ was measured in the absence and presence of chlorpromazine (CPZ), nystatin (NYS), amiloride (AML), and their combinations (CPZ+NYS, CPZ+AML, and NYS+AML). The Papp (×10^−5^ cm/s) values for 200 μg/mL PHP_0.5MSC-F_ in the AP→BL direction at 30 min, 60 min, and 120 min were measured. Notably, the differences in the Papp values when the endocytic modulator was present vs. absent were not found to be statistically significant (*p* > 0.05) for PHP_0.5MSC-F_ (Figure 6A). However, when the Papp (×10^−5^ cm/s) values for 200 μg/mL PHP_H-1-2_ were measured in the AP→BL direction at 30 min, 60 min, and 120 min, they were significantly lower in the presence of the endocytic modulators (*p* < 0.01) (Figure 6B).

## 3. Discussion

We previously determined that the PHP polysaccharide content using a phenol-sulphuric acid colorimetric analysis method with D-glucose as the standard [9]. PHP_H-1-2_ was a glucan which only composed of glucose monosaccharide, the content of glucose was 97.20%. PHP_0.5MSC-F_ was a heteroglycan which composed of rhamnose, galactose, arabinose, glucose and galacturonicacid, the monosaccharide content of glucose was 26.63%. This method is used widely because of its sensitivity, convenience, and practicability. Coexisting amino acids and proteins can interfere with the measurements, and complex biological sample matrices can result in a multitude of side reactions. Therefore, this method would not be appropriate for measuring the polysaccharide content in blood, bodily fluid, or cell-culture media. The methylene group of PMP can undergo a condensation reaction with the aldehyde or acetal groups of the reducing sugars. Thus, the addition of the fluorescence group can produce PHP sugar-PMP derivatives that can be analyzed with HPLC-UV methods. Except for fructose, sugar alcohols, and nonreducing sugars, simple sugars can be quantified using the PMP derivatization method, which has the advantages of a low limit of detection, high sensitivity, and good selectivity. Through this method, polysaccharides were subjected to complete hydrolysis with acid and converted into simple sugars. Then, the monosaccharide composition and content were analyzed using GC, HPLC, and GC-MS. The PMP derivatization method is not suited for quantifying complete polysaccharides in biological samples.

In this study, two PHP polysaccharides, PHP_0.5MSC-F_ and PHP_H-1-2_, were labeled with 2-AB to develop a novel fluorescent probe for HPLC-FLD. The detection limit for PHP_0.5MSC-F_-2-AB was found to be 8.84 μg/mL with a linear range of 29.45–683.28 μg/mL. The detection limit for PHP_H-1-2_-2-AB was found to be 13.89 μg/mL with a linear range of 46.29–462.76 μg/mL. The method showed little interference and high sensitivity and was able to determine whether the full polysaccharide molecule was present in the cell-culture medium.

Caco-2 cell monolayers serve as a model for forecasting drug absorption and transport and provide a quick and simple means for estimating drug bioavailability. The bidirectional transport of polysaccharides in the apical to basolateral (AP→BL) and basolateral to apical (BL→AP) directions was measured by HPLC-FLD with 2-AB tags. The bidirectional transport evaluations of PHP_0.5MSC-F_ and PHP_H-1-2_, in which the AP→BL Papp values were greater than the BL→AP Papp values, suggested the occurrence of passive transference.

The AP→BL transport of PHP_0.5MSC-F_ and PHP_H-1-2_ in the absence and presence of the verapamil (P-gp inhibitor) revealed no significant difference (*p* > 0.05) in Papp values. This finding reveals that the efflux of PHP_0.5MSC-F_ and PHP_H-1-2_ is not regulated by P-glycoprotein (P-gp). Notably, both polysaccharides showed good membrane permeability and were not identified as P-gp efflux transporter substrates.

Chlorpromazine is a clathrin inhibitor, nystatin is a caveolin inhibitor, and amiloride is a macropinocytosis inhibitor. To explore the endocytosis pathways of the two polysaccharides in caco-2 cells, the cells were cultured with CPZ, NYS, AML, CPZ+NYS, CPZ+AML, and NYS+AML, and the resulting Papp values were measured to determine whether clathrin-mediated endocytosis, caveolin-mediated endocytosis, or micropinocytosis play roles in polysaccharide internalization. The AP→BL transport evaluation of PHP_0.5MSC-F_ in the absence and presence of different endocytosis inhibitors revealed no significant differences (*p* > 0.05) in Papp values. Thus, the absorption mechanism of PHP_0.5MSC-F_ might merely comply with passive diffusion under a concentration gradient. However, the uptake experiment performed with 400 mg/mL PHP_H-1-2_ for 2 h at 37 °C revealed a Papp decline of 46.99% in the presence of CPZ. Notably, in the presence of NYS, AML, CPZ+NYS, CPZ+AML, and NYS+AML, the Papp values also declined by 45.74%, 45.39%, 51.66%, 47.22%, and 38.20%, respectively. Thus, the absorption mechanism of PHP_H-1-2_ may involve multimodal endocytosis pathways mediated by chlorpromazine, nystatin, and amiloride. When the three inhibitors were used singly, the chlorpromazine-treated cells absorbed the least polysaccharide. When two of the three inhibitors were used in combination, the chlorpromazine-containing groups absorbed the least polysaccharide. Thus, PHP_H-1-2_ mainly utilized clathrin-mediated endocytosis pathways in caco-2 cells.

## 4. Materials and Methods

### 4.1. Instruments

The high performance liquid chromatography (HPLC) system (model 2996) was equipped with a 2475 fluorescence detector (FLD), an auto-sampler, and ultrahydrogel TM500/1000(7.8 × 300 mm)GPC columns manufactured by Waters Technologies (Milford, MA, USA). The LC-20A high performance liquid chromatography instrument was equipped with a RF-10AXL fluorescence detector produced by Shimadzu (Japan). The HiTrap desalting column (model 71-7154-00 AK) was produced by GE Healthcare (Little Chalfont, Buckinghamshire, UK). The hypersil C_18_ column (4.6 mm × 250 mm, 5 μm) was made by Dalian Elite Analytical Instruments Co., Ltd. (Dalian, China). The superclean bench (model SW-CJ-1FD) was produced by Suzhou Purification Equipment Co., Ltd. (Suzhou, China). The CO_2_ training box (model HERAcell 150i) was produced by Thermo Fisher Scientific (Shanghai, China). The fluorescent inverted microscope (model IX51) was produced by Olympu (Tokyo, Japan). MillicellRES-2 Volt-Ohm meter and accessories produced by Millipore (Bedford, MA, USA) were used.

### 4.2. Chemicals

The 2-AB glycan labeling kit was purchased from Ludger Ltd. (Oxfordshire, UK). Sugar standards, NaBH_3_CN, tetra-n-butylammonium fluoride (TBAF), and dimethylsulfoxide (DMSO) were purchased from Sigma–Aldrich (Shanghai, China). Dextran T-500, T-70, T-40, and T-10 were procured from Pharmacia (Stockholm, Sweden). Penicillin/streptomycin solution, 0.25% trypsin-EDTA, RPMI-1640, and PBS were purchased from HyClone Products (Logan, UT, USA). GIBCO fetal bovine serum (FBS) was obtained from Invitrogen (Chicago, CA, USA). Water was deionized using the Milli-Q-Plus ultra-pure water system (Milford, MA, USA).

### 4.3. Fluorescent Labeling of Polysaccharides with 2-Aminobenzamide

Dextran standard-2-AB preparation: 350 mg of TBAF was dissolved in 350 μL of DMSO, to which 120 μL HAC was added. After mixing, 5 mg quantities of dextran T-500, T-70, T-40, and T-10 were added, and the sample was vortexed for 30 min and allowed to dissolve for 1 h at 65 °C until the solution clarified. Subsequently, 2-AB (25 mg) and NaBH_3_CN (30 mg) were added and reacted for 4 h at 65 °C.

The procedures used for the preparation of PHP_H-1-2_-2-AB and the dextran standard-2-AB were identical.

PHP_0.5MSC-F_-2-AB preparation: 350 mg of TBAF was dissolved in 350 μL of DMSO, to which 120 μL of HAC was added. After mixing, 5 mg of polysaccharide 0.5MSC-F was added, and the solution was vortexed for 30 min and then dissolved for 1 h at 65 °C until the solution clarified. Subsequently, 2-AB (20 mg) and NaBH_3_CN (10 mg) were added and reacted for 4 h at 65 °C.

Fluorescent labeling of polysaccharides for purification: Once the reaction was complete, 3.5 mL of absolute alcohol was added to the solution for fluorescence quenching, and the reaction was terminated through centrifugation for 10 min at 3000 rpm; the supernatant was subsequently removed. The products of this reaction settled at the bottom of the container and were resuspended in 15 mL of distilled water. The precipitate was washed three times until 2-AB was not detected in the supernatant. The fluorescently labeled polysaccharides (polysaccharide-2-AB) were obtained by freeze-drying.

### 4.4. Polysaccharide-2-AB Analysis by Thin Layer Chromatography

To test the fluorescent polysaccharide purity, the labeling reaction mixtures, with or without 2-AB, were directly evaluated using TLC. Silica gel G TLC plates (Merck) were run with propanol-aqueous (3/1, *v/v*) as the developing solvent.

A pencil was used to draw a line of about 1 cm from the bottom of the TLC plate. A microcapillary tube, 0.3 mm in diameter, was used to apply 15 μL of sample to the line, and the sample was allowed to completely dry. About 1 cm of propanol-aqueous (3/1, *v/v*) developing agent was poured into the developing chromatography tank bottom. The TLC plates were placed in the chromatographic tank as close to vertical as possible. The lid was closed tightly, and chromatography was allowed to proceed until the solvent reached a level of about 1 cm from the upper end. Then, the plates were removed from the chamber and allowed to dry on a thin-layer heater. Deionized water was used to rinse the TLC plates using the same procedure. After repeating three times, the excess reactants were removed almost completely.

### 4.5. Polysaccharide Purification, Content and Molecular Weight Determination

A Shimadzu LC-20A high performance liquid chromatography instrument equipped with a RF-10AXL fluorescence detector and an Ultrahydrogel ^TM^ 1000 gel chromatographic column (78 mm × 300 mm) was used. The mobile phase was 0.1 mol/L NaNO_3_, with a flow rate of 0.5 mL/min. In the phenol-sulphuric acid method, 0.05 mL of 80% phenol was added to 1 mL of polysaccharide solution followed by 5 mL of sulphuric acid with mixing. The solution reacted at 85 °C temperature within 15 min and cooled to room temperature, the absorbance was detected. Then the absorbance was calculated as concentration of PHP from working curve of D-glucose. Dextran-2-AB samples (T10-2-AB, T40-2-AB, T70-2-AB, and T500-2-AB) were used as references, from which the average molecular weights (MWs) and purity levels of PHP_H-1-2_-2-AB and PHP_0.5MSC-F_-2-AB were determined.

### 4.6. Polysaccharide-2-AB Analysis by HPLC-FLD

The HPLC analysis was carried out using a Hypersil C_18_ column. The mobile phase, flow rates, and column temperature were optimized to establish the HPLC method. The mobile phase contained only 0.1 mol/L NaNO_3_ aqueous solution with an isocratic elution flow rate of 0.5 mL/min. The fluorescence detector wavelengths were set to an excitation wavelength (λ_ex_) of 320 nm and an emission wavelength (λ_em_) of 420 nm, and the column temperature was set to 35 °C. The PHP_0.5MSC-F_-2-AB, PHP_H-1-2_-2-AB, and reference dextran (T-500, T-70, T-40, and T-10) samples were all prepared at a concentration of 240 μg/mL with a sample size of 20 μL.

### 4.7. Cell Culture

Caco-2 cells (human colon adenocarcinoma) were purchased from Shanghai Institutes for Biological Sciences (Shanghai, China), ATCC No. HTB-37^TM^. Caco-2 cells were cultured in RPMI-1640 medium containing 20% FBS, 100 μg/mL of streptomycin, and 100 units/mL penicillin at 37 °C in an incubator with 5% of CO_2_. The culture medium was changed the next day, and the cells were passaged every four days.

Cells from passages 30–40, at a concentration of 2 × 10^5^ cells/mL, were added to 24-well transwell plates coated with a polycarbon ester membrane.

Cell culture medium was added to the AP (0.5 mL/well) and BL (1.5 mL/well) sides and replaced every two days for the first week. The culture medium was changed every day on the AP side after eight days and every two days on the BP side until 21 days. The transmembrane electrical resistance (TEER) value of the caco-2 cell monolayer was measured using a MillicellRES-2 Volt-Ohm meter with a mode STX01 electrode when the TEER value was more than 400 Ω·cm^2^ for transportation studies.

### 4.8. Bidirectional Polysaccharides Transport Tests

The caco-2 cell monolayers were rinsed twice with HBSS (Hanks balanced salt solution, pH 7.4) and incubated with fresh HBSS for 30 min at 37 °C in 5% CO_2_. Then, to examine polysaccharide absorption and transport from the AP to the BL surfaces, the HBSS was removed and replaced with 0.5 mL of polysaccharide on the AP side, and 1.0 mL of HBSS was added to the BL side. To examine the BL to AP direction, 1.0 mL of polysaccharide was added to the BL supply side, and 0.5 mL of HBSS was added to the AL receiving side. After incubation at 37 °C in 5% CO_2_ for 30–120 min, the polysaccharide concentrations on the BL and AP sides were determined using FLD-HPLC. The polysaccharide caco-2 cell accumulation and apparent permeability coefficient were calculated.

### 4.9. The Influences of Verapamil, Chlorpromazine, Nystatin, and Amiloride on Polysaccharide Transport, Determined by FLD-HPLC

The caco-2 cells were first cultured with 100 μmol/L of verapami [25], 20 mg/mL of chlorpromazine, 15 mg/mL of nystatin, 550 mmol/L of amiloride, and two of the following three agents (chlorpromazine, nystatin, and amiloride) for 1 h at 37 °C. Then, an uptake experiment was performed with polysaccharides in the presence of different endocytosis inhibitors for 2 h at 37 °C [26]. The influences of verapamil, chlorpromazine, nystatin, and amiloride on polysaccharide transport were estimated.

The caco-2 cells were first cultured with 100 μmol/L of verapamil, 20 mg/mL of chlorpromazine, 15 mg/mL of nystatin, and 550 mmol/L of amiloride, as well as two of the three agents chlorpromazine, nystatin, and amiloride for 1 h at 37 °C. Then, an uptake experiment was performed with polysaccharides in the presence of different endocytosis inhibitors for 2 h at 37 °C [26]. The influences of Verapamil, Chlorpromazine, Nystatin and Amiloride on the transport of polysaccharides were observed.

### 4.10. Statistical Analyses

All data were treated using SPSS for statistical analyses. All values are expressed as the mean ± SD while *p* < 0.05 indicated a significant difference.

## 5. Conclusions

In this study, fluorescent 2-AB was used to explore the bidirectional transport mechanism of *Pseudostellaria heterophylla* polysaccharides across caco-2 cells. PHP_0.5MSC-F_ and PHP_H-1-2_ mainly underwent passive transference. The two polysaccharides were not P-gp efflux transporter substrates. The absorption mechanism of PHP_0.5MSC-F_ is suggested to be passive diffusion under a concentration gradient, whereas PHP_H-1-2_ mainly utilizes a clathrin-mediated endocytic pathway to enter caco-2 cells. This is the first time fluorescent labeling has been used to investigate intestinal epithelial cell absorption and transport of dextran and pectin polysaccharides from *Pseudostellaria heterophylla*. This innovative HPLC-FLD method can help to track polysaccharide internalization in vitro and in vivo to facilitate cellular uptake and biodistribution exploration.

## Figures and Tables

**Table 1:** RT and average molecular weight of dextran standards and polysaccharide samples with 2-AB fluorescence labeling.

Data	PHP_0.5MSC-F_	PHP_H-1-2_	T10	T40	T70	T500
Rt (min)	18.03	16.49	19.89	18.22	17.45	15.13
logM	4.6661	5.2089	4.0000	4.6021	4.8451	5.6990
M_W_ (Da)	46,355	161,770	10,000	40,000	70,000	500,000

**Table 2:** Accumulative transport amount and apparent permeability coefficient of PHP_0.5MSC-F_ and PHP_H-1-2_ in the caco-2 monolayer.

Polysaccharides	Concentration (μg/mL)	Transport (min)	Accumulative Transport Amount (μg/mL)		Apparent Permeability Coefficient (×10^−5^ cm/s)	
			AP→BL	BL→AP	AP→BL	BL→AP
PHP_0.5MSC-F_	100	30	13.88 ± 1.93	2.50 ± 0.29	23.11	4.19
		60	17.45 ± 2.11	3.35 ± 0.16	14.61	2.81
		120	20.61 ± 2.10	5.84 ± 0.33	8.63	2.43
	200	30	37.43 ± 2.62	8.11 ± 0.83	31.32	6.78
		60	51.76 ± 3.16	8.73 ± 0.26	21.65	3.64
		120	66.90 ± 3.69	12.15 ± 1.32	14.01	2.53
	400	30	82.00 ± 4.28	15.20 ± 2.62	34.33	6.36
		60	109.2 ± 4.78	20.40 ± 2.41	22.86	4.27
		120	171.8 ± 6.27	22.64 ± 3.05	17.98	2.37
PHP_H-1-2_	100	30	25.34 ± 2.09	3.51 ± 0.17	42.37	5.86
		60	32.22 ± 2.62	5.13 ± 1.27	26.97	4.27
		120	35.83 ± 3.32	6.85 ± 1.32	14.99	2.87
	200	30	53.51 ± 4.41	8.90 ± 1.10	44.8	7.45
		60	63.80 ± 4.05	10.42 ± 1.69	26.71	4.35
		120	70.63 ± 4.73	13.35 ± 2.78	14.78	2.78
	400	30	111.6 ± 4.16	20.19 ± 2.62	46.73	8.46
		60	128.2 ± 5.83	30.18 ± 3.16	26.84	6.32
		120	173.4 ± 6.41	40.20 ± 4.83	18.67	4.21

## Data Availability

The data presented in this study are available on request from the corresponding author.
